# Ballistic thermal transport in silicon nanowires

**DOI:** 10.1038/srep41794

**Published:** 2017-02-02

**Authors:** Jeremie Maire, Roman Anufriev, Masahiro Nomura

**Affiliations:** 1 Institute of Industrial Science, The University of Tokyo, Tokyo 153-8505, Japan; 2 Laboratory for Integrated Micro Mechatronic Systems/National Center for Scientific Research-Institute of Industrial Science (LIMMS/CNRS-IIS), The University of Tokyo, Tokyo 153-8505, Japan; 3 Institute for Nano Quantum Information Electronics, The University of Tokyo, Tokyo 153-8505, Japan; 4 PRESTO, Japan Science and Technology Agency, Saitama 332-0012, Japan

## Abstract

We have experimentally investigated the impact of dimensions and temperature on the thermal conductivity of silicon nanowires fabricated using a top-down approach. Both the width and temperature dependences of thermal conductivity agree with those in the existing literature. The length dependence of thermal conductivity exhibits a transition from semi-ballistic thermal phonon transport at 4 K to fully diffusive transport at room temperature. We additionally calculated the phonon dispersion in these structures in the framework of the theory of elasticity and showed that the thermal conductance increases with width. This agrees with our experimental observations and supports the pertinence of using the modified phonon dispersion at low temperatures.

Nanoscale heat transport has been attracting interest due to its importance and expected applications in fields such as electronic chip heat management and thermoelectric energy harvesting. This interest has led to numerous studies of the electrical^1^,^2^, optical^3^,^4^ and thermal properties^5^,^6^,^7^ of nanowires (NWs). The combination of good electrical and poor thermal conductivity makes NWs suitable candidates for thermoelectric applications^7^,^8^,^9^,^10^. The reduction of thermal conductivity typically occurs due to phonon scattering at the boundaries, e.g. oxide shell structures^11^,^12^,^13^, the interface between regions of superlattices^14^,^15^,^16^ or just rough boundaries^17^,^18^,^19^. Such surface scattering is usually diffuse, i.e. the momentum vectors of incoming phonons change upon scattering, both in intensity and orientation, in contrast to specular surface scattering where both are preserved. Specular phonon scattering leads to non-diffusive heat propagation called “ballistic”, meaning the absence of scattering mechanisms other than specular phonon reflections on the surfaces. Such ballistic heat transport has been demonstrated at room temperature in bulk^20^,^21^ and in suspended silicon thin films^22^. In addition, it has been demonstrated in SiGe alloy NWs^23^ and Si-Ge core-shell NWs^24^ where the mean free path of phonons is much longer than that in Si^25^. It was found that in NWs shorter than 8.3 μm, heat conduction was purely ballistic even at room temperature. Since then, due to the stringent requirements for the smoothness of surfaces, there has been no other report of ballistic conduction in NWs. The interest in ballistic transport lies in the fact that it is a required property for phonon interference to occur in periodic nanostructures^26^, which allows the coherent control of heat transport. Thus, it is of crucial importance to know the conditions under which specular scattering, and thus ballistic transport, can occur in structures fabricated by a top-down approach.

In this work, we focus on Si NWs fabricated via a top-down approach. Although their surface cannot yet be as flat as that of as-grown NWs, integration in devices is expected to prove more convenient. First, we study the thermal transport in NWs of different widths at temperatures in the 10–300 K range, and observe good agreement with the literature. Next, we demonstrate the ballistic heat transport in short nanowires at 4 K. Finally, we address the modifications to the phonon dispersion in these NWs and show that they can explain the stronger reduction in thermal conductivity of NWs at 4 K, as compared to that of the membrane. These results demonstrate the possibility of using phonon wave properties to control heat transport at the nanoscale.

## Fabrication and Measurements

**Figure 1:**
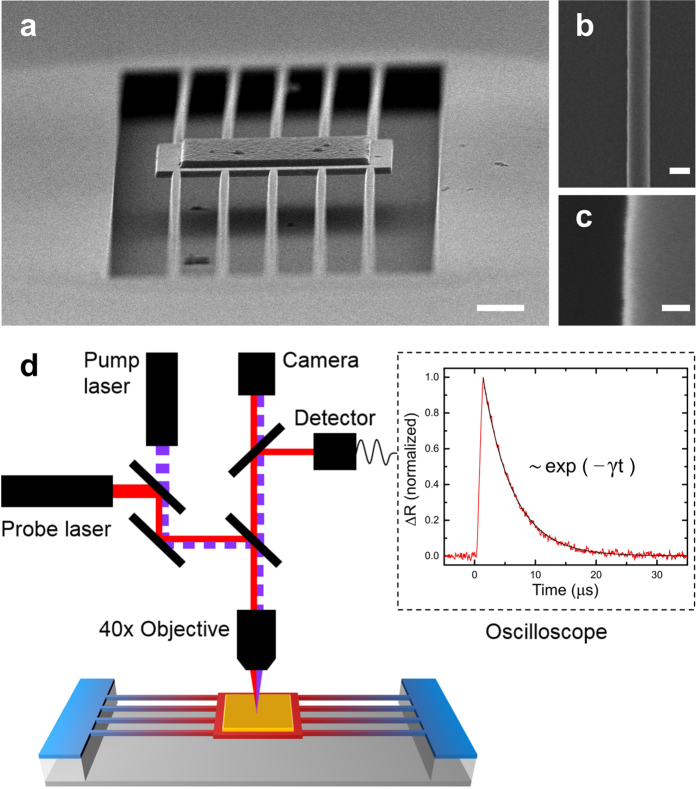
Experimental sample and characterization platform. (**a**) SEM image of a typical sample, (**b**) single NW, and (**c**) NW side wall; scale bars are 1 μm, 100 and 10 nm, respectively. (**d**) Simplified schematic of the thermoreflectance measurement setup. A typical signal, recorded by the oscilloscope, is shown with its corresponding exponential decay.

All samples were fabricated using a standard top-down approach on a (100) silicon-on-insulator wafer with a 145 nm-thick upper single-crystalline silicon layer. Al pads, 4 × 4 μm in area and 125 nm in thickness, were patterned by electron beam (EB) lithography and then deposited via electron beam-assisted metal evaporation. The NWs were shaped by EB lithography around the existing Al pads followed by a transfer to the silicon layer using inductively coupled plasma reactive-ion etching. The buried oxide layer was subsequently removed with hydrofluoric acid vapour to suspend the structures. A scanning electron microscope (SEM) image of the complete suspended structure is shown in Fig. 1a.

We fabricated samples with NW widths (w) ranging from 88 to 122 nm and lengths ranging from 0.5 to 7 μm. The NW thickness was 145 nm for all samples. A close-up view of a NW is shown in Fig. 1b and a magnified image of the surface quality is displayed in Fig. 1c. The surface roughness was estimated to be about 2 nm.

To measure the in-plane thermal conductivity, we used a micro time-domain thermoreflectance (μ-TDTR) system. A schematic of the experimental setup is shown in Fig. 1d. The pulsed pump (642 nm) and continuous probe (785 nm) laser beams are focused through a 40× microscope objective with a numerical aperture of 0.6 onto the Al pad, which serves as a heater and sensor. The reflected probe beam is detected by a Si photodiode with a bandwidth of 200 MHz. The heat provided by the lasers to the Al pad can dissipate only through the NWs. Unlike in the electrical measurements, the NWs are not bonded to the heater but directly integrated in the Si layer. The measurement output is proportional to temperature and reveals an exponentially decaying trend *exp*(−*γt*), with *γ* as the decay rate. An example of a typical signal with its corresponding exponential decay is shown in Fig. 1d. The decay rate (*γ*) is then input in a 3D finite elements method model, which virtually reproduces the experiment to extract the thermal conductivities. The error in the thermal conductivity measurements is estimated by considering the error on the measurement of dimensions by the SEM and the error in the measurement of the decay rate. The value of *γ* is measured continuously and recorded when the standard deviation over the last 30 iterations is less than 2%. The error in the measurement of the width of the nanowires is typically 2–4 nm. The impact of these errors on the determination of thermal conductivity is estimated^27^ to be less than 5%, which is the value considered in this work in the absence of any indication. Further details about the measurement system and error analysis can be found elsewhere^27^,^28^,^29^.

## Results

**Figure 2:**
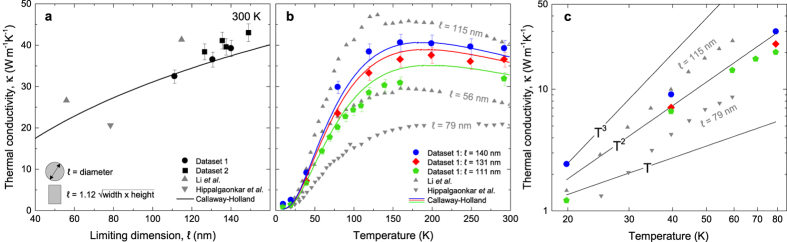
Dimension and temperature dependence of NW thermal conductivity. (**a**) Thermal conductivity of two Si NW datasets as a function of the limiting dimension (ℓ) at 300 K, together with the data by Li *et al*.^32^ (upwards grey triangles) and Hippalgaonkar *et al*.^30^ (downwards grey triangle), and the prediction of the Callaway**–**Holland model (line). (**b**) Temperature dependence of the thermal conductivity for these NWs. (**c**) Low temperature thermal conductivities follow a T^2^ trend up to 80 K. Data from Li *et al*. and Hippalgaonkar *et al*. follow a T^3^ and T^2.5^ trend, respectively.

First, we studied the dependence of thermal conductivity as a function of NW width, as the literature data on NWs fabricated by a top-down approach are scarce^30^. We measured the thermal transport in NWs with constant thickness (h = 145 nm) and widths (w) of 68, 94, 101, 102, 108, and 122 nm, fabricated on three different chips from the same wafer. To compare our data to the literature, we used the limiting dimension, defined as 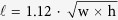
31 for NWs with a rectangular cross-section and where 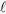 is the diameter for circular NWs. The results, displayed in Fig. 2a together with the experimental data obtained by Li *et al*.^32^ and Hippalgaonkar *et al*.^30^, show that the conductivity increases with the limiting dimension. This is to be expected^27^,^33^ as the number of surface scattering events is lower in wider NWs. The increase with width agrees with our previous measurements on nanowires in tendency, but the absolute values were overestimated in that work^34^. In addition, we plotted the thermal conductivities (*κ*) predicted by the Callaway–Holland model^35^, described by the expression:


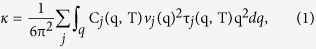


where j is the number of the phonon mode, ω(q) is the phonon dispersion in bulk, C(q, T) is the heat capacity at temperature T, *v* is the phonon velocity, and τ the total phonon lifetime. This lifetime was calculated using Mathiessen’s rule with the expressions of lifetimes for various scattering mechanisms being 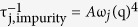, 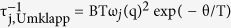, and 
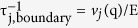
 for impurity, Umklapp, and boundary scattering, respectively, in which θ is the reduced Debye temperature and A, B, and E are the fitting parameters. First, by fitting the thermal conductivity data of the bulk, we obtained the following parameters: A = 2.95 × 10^−45^ s^3^, B = 0.95 × 10^−19^ s·K^−1^, and E = 3.3 × 10^−3^ m. Then, by fixing the parameters A and B, we calculated the thermal conductivity as a function of the parameter E, which stands for the limiting dimension. Figure 2a shows that our measurements follow the same trend as the Callaway–Holland model. Further measurements were then performed on three of these structures at temperatures between 10 and 300 K, and the results are presented in Fig. 2b together with the data from Li *et al*.^32^ and Hippalgaonkar *et al*.^30^ The thermal conductivities of these different datasets are all in good agreement: wider NWs display a higher thermal conductivity in the entire temperature range. The peaks of the temperature dependencies are shifted to higher temperatures than that of bulk Si (25 K) and depend on the limiting dimension of the NWs (i.e. 160 K for 111 nm; 200 K for 131 nm), suggesting that the phonon mean free path is limited by boundary scattering rather than Umklapp scattering^17^. At low temperatures, as shown in Fig. 2c, the data from Li *et al*. display a T^3^ trend, as expected from the Debye theory for bulk^32^. Hochbaum *et al*.^17^ completed this dataset with NWs of similar diameters but with significant roughness (σ = 1–5 nm). They demonstrated how surface roughness could increase the role of surface phonon scattering. We can see that our measurements follow a T^2^ trend, which was also observed by Li *et al*. for thinner wires and Hochbaum *et al*. for NWs with diameters of 115 and 98 nm (not shown). As our fabrication method introduces a surface roughness of about 2 nm, our T^2^ observation is consistent with the data from Hochbaum *et al*.

The Callaway–Holland model was then used to fit the measured temperature dependencies. The boundary scattering term (E) is the only adjustable parameter, and is set equal to the limiting dimension. The experimental results agree with the simulations (lines) over the whole range of temperatures, as shown in Fig. 2b, suggesting diffusive transport. These results also indicate that the thermal conductivity of NWs exhibits similar values, regardless of the fabrication method, as long as the limiting dimensions are identical. Nonetheless, typically lower surface roughness in the bottom-up fabrication method results in the slightly higher thermal conductivity of NWs by Li *et al*. as compared to our data. Conversely, nanowires by Hippalgaonkar *et al*. have a significantly lower thermal conductivity, which stems from much a larger surface roughness^30^, combined with a smaller minimum dimension.

While no ballistic phonon transport has been reported for Si NWs at room temperature so far, some thermal conductance measurements below 5 K could not be fully explained by a diffusive model alone^36^,^37^. To detect the presence of ballistic heat transport, the most straightforward approach is to measure the thermal conductivities of NWs with different lengths. Indeed, in the diffusive heat transport regime, the thermal conductance of the system is proportional to the ratio of the NW cross-section to its length. However, in the ballistic heat transport regime, the conductance is independent of the length, as neither the surfaces nor internal processes introduce additional thermal resistance. Thus, the conductivity will linearly increase with length.

**Figure 3:**
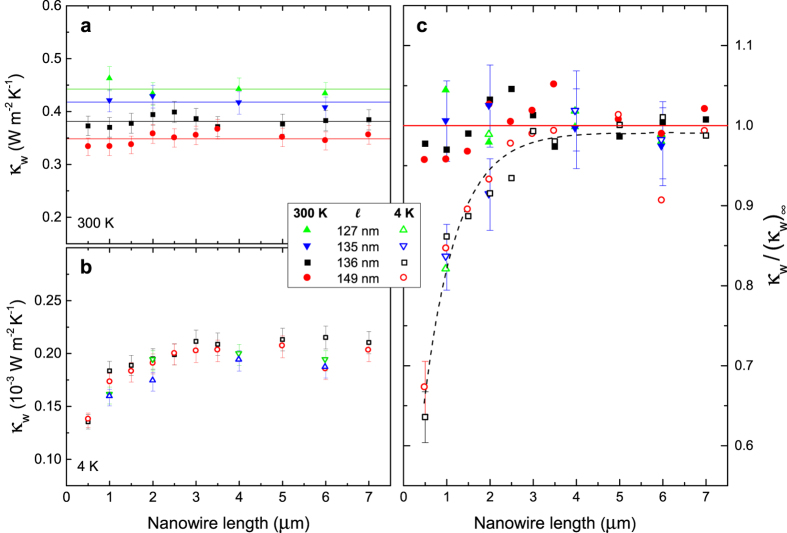
Length dependence of NW thermal conductivity. Thermal conductivity of NWs, normalized by the width (*κ*_w_ = *κ*/w) at (**a**) 300 K and (**b**) 4 K. (**c**) *κ*_w_ normalized to its value for long NWs (>4 μm) at 300 K and 4 K. The red and black lines are visual guides and correspond to the value 1 and an exponential fit, respectively.

Therefore, we measured NWs with lengths between 0.5 and 7 μm. As our NWs have a surface roughness of about 2 nm, comparable to the dominant phonon wavelength at room temperature^38^, surface scattering at room temperature is expected to be mostly diffuse^39^. For this reason, room temperature thermal conductivity of these NWs is independent of the length (Fig. 3a).

At 4 K, however, a clear difference between short and long NWs appears (Fig. 3b): in NWs longer than 4 μm, the thermal conductivity is still independent of the length, but in NWs shorter than 4 μm, the thermal conductivity increases with length, which is characteristic of the ballistic transport regime^23^,^24^. For a convenient comparison, data at both 300 K and 4 K are presented in Fig. 3c, normalized to that of the longest NW. Despite this, although usually neglected, it has been suggested that the contact resistance may falsify such an interpretation^40^. We thus calculate the contact resistance. First, we linearly fit the total resistance as a function of length for the data in the diffusive regime – constant thermal conductivity – between 3 and 6 μm. The intercept of this fit is the contact resistance, and its value is at least one order of magnitude smaller than the smallest NW resistance, measured at 4 × 10^9^ KW^−1^. Thus, the contact thermal resistance is negligible, as expected in the case of a monolithic contact between the nanowires and the rest of the device^30^. The length-dependent thermal conductivity is thus attributed to ballisticity. The fact that ballisticity is not indefinitely preserved shows that either the surface scattering is only partially specular or the internal scattering processes limit the mean free path. To separate the contribution of surfaces to the reduction in thermal conductivity from that of internal processes, we calculate the thermal conductivities (*κ*) of the NWs normalized by their width (w)^41^ *κ*_w_ = *κ*/w as displayed in Fig. 3a and b for temperatures of 300 K and 4 K, respectively. Although the thermal conductivity is not expected to be linearly proportional to the width, the relatively small variation in the limiting dimension legitimizes this assumption. The normalized conductivity decreases as the NW width is increased. This suggests that scattering mechanisms other than the surfaces – impurity and Umklapp scattering – play a larger role in the larger NWs at room temperature. This can be explained by the fact that in wider NWs, phonons can travel long enough between surfaces to be scattered more often by these mechanisms. At 4 K, however, all data points were perfectly matched with each other, which indicates that the only scattering mechanism influencing thermal conductivity at 4 K is surface scattering. Furthermore, this surface scattering is only partially specular, as perfect specularity would make the absolute value of thermal conductivity independent of the NW width. Remarkably (see Fig. 3a and c), even at 300 K, NWs with a length of 0.5 μm seem to have a lower thermal conductivity, which could be a sign of semi-ballisticity, even at room temperature.

## Discussion

**Figure 4:**
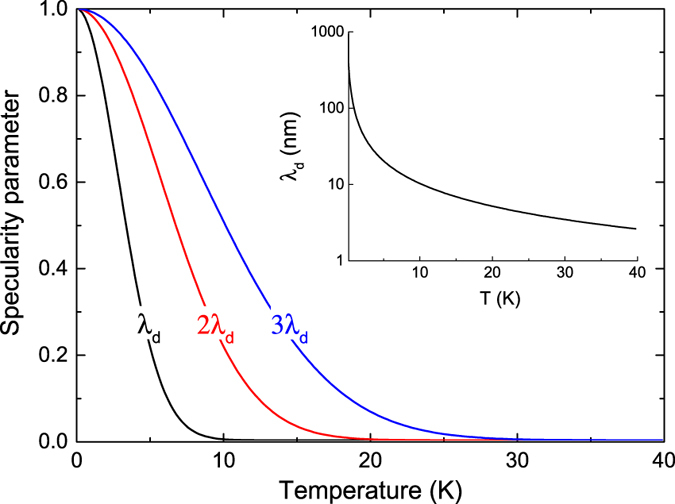
Specular scattering probability. Probability of specular surface scattering as a function of temperature for the dominant wavelength *λ*_d_ and two longer wavelengths (2*λ*_d_ and 3*λ*_d_). Angles of incidence of phonons are considered isotropic. (inset) Dominant wavelength as a function of temperature.

The dominant phonon wavelength calculated via the Debye theory^31^,^42^ at 4 K is 25.5 nm, which is well above its value at room temperature (a few nanometres) and thus well above the surface roughness. The specularity parameter, or probability of specular reflection (*p*) from the side walls is given by^39^:


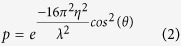


where *η* is the mean surface roughness (±2 nm at the side walls and ≤0.5 nm at the top and bottom surfaces), *λ* is the wavelength, and *θ* is the angle of incidence of phonons on the surface. Assuming isotropic angles of incidence, the value of *p*, obtained for the dominant wavelength of 25.5 nm at 4 K, is 0.38 at the side walls and about 1 at the top and bottom surfaces. This is illustrated in Fig. 4, where the specularity is plotted as a function of temperature for the dominant wavelength (*λ*_*d*_), alongside two longer wavelengths (2 × *λ*_*d*_ and 3 × *λ*_*d*_). Owing to the Bose–Einstein distribution, phonons with longer wavelengths will also contribute to heat transport and their probability of specular reflection will be higher than that of the dominant wavelength, regardless of the temperature, as shown in Fig. 4. The dominant wavelength *λ*_*d*_, shown in the inset of Fig. 4, decreases as 1/T.

This partial specularity leads to a semi-ballistic heat transport. The transition to the purely diffusive regime in long NWs occurs when the length of the system becomes longer than the phonon mean free path, i.e. the distance that phonons can travel without internal or diffuse surface scattering. While the mean free paths in bulk are well above several microns at low temperature^43^, diffuse surface scattering limits its value in NWs.

**Figure 5:**
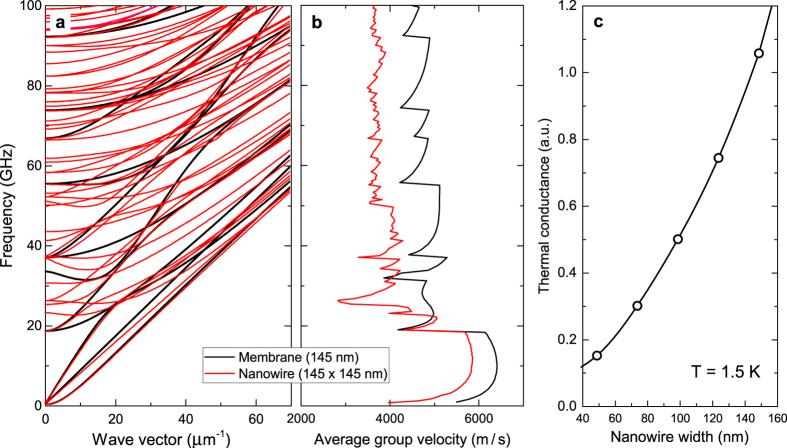
Phonon dispersion properties in NWs. (**a**) Phonon dispersion of a 145-nm-thick membrane (black) and a square NW (145 × 145 nm) (red). (**b**) Group velocity spectra extracted from the phonon dispersion for both the membrane and NW. (**c**) Thermal conductance calculated for NWs of different width at 1.5 K.

When surface scattering is specular, phonons can interfere and it is appropriate to consider that phonons follow the modified dispersion relation^26^ in NWs. We compared the phonon dispersion of a 145-nm-thick membrane and square NWs (145 × 145 nm^2^) using a finite elements model based on the linear theory of elasticity^44^ (Fig. 5a). The dispersion of the NW possesses a higher density of states, as the number of bands is larger than that of the membrane. However, the impact on thermal conduction cannot be assessed by considering the dispersion only. For that reason, we additionally calculated the group velocity spectrum (Fig. 5b) and thermal conductance^45^ (Fig. 5c). The group velocity of phonons, which is included in the expression of the thermal conductance, is about 20% lower in NWs of width 145 nm than it is in the membrane. The thermal conductance has been calculated for NWs with widths ranging from 50 to 150 nm at 1.5 K. Although simulations were performed only at 1.5 K, we expect nearly the same trend at 4 K because the data converge with the increase in temperature, and the tendency barely changes above 1 K. The width dependence of thermal conductance (Fig. 5c) is attributed to both the varying cross-section and the modified phonon dispersion. Indeed, as the width decreases, the decrease in thermal conductance is more than twice as strong as the reduction in the cross section, showing that changes in the dispersion have a strong impact. The absence of any visible width dependency in Fig. 3c stems from the following: (i) the normalization performed on the thermal conductivity and (ii) the fact that only part of the phonon spectrum is ballistic in our experiments at 4 K.

**Figure 6:**
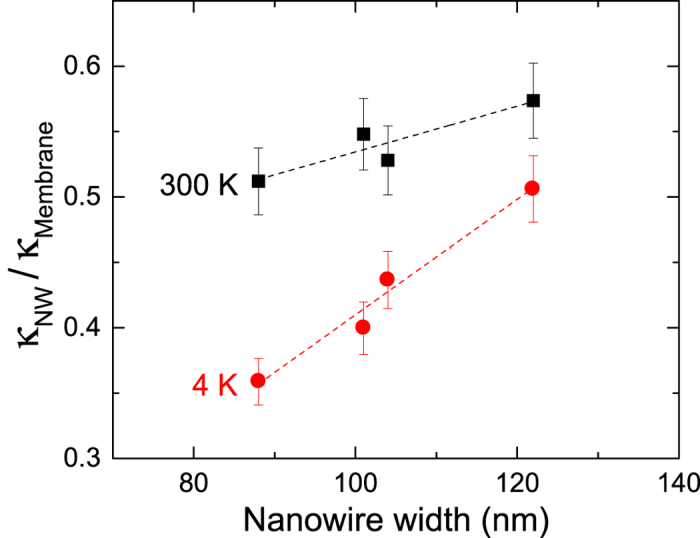
Relative conductivities as a function of width. *κ*_*nw*_/*κ*_*membrane*_ measured experimentally at 300 K (squares) and 4 K (circles).

At 300 K, the boundary scattering is diffusive, and thus the NW phonon dispersion cannot be used as the phase is destroyed upon scattering on the surface. At 4 K however, since boundary scattering is specular, the modified phonon dispersion of NWs is applicable. In this case, it is expected that the values of the relative thermal conductivity *κ*_*nw*_/*κ*_*membrane*_ should be lower at 4 K than at 300 K. In Fig. 6, we plotted the reduced thermal conductivity obtained experimentally. Regardless of the width, the values of *κ*_*nw*_/*κ*_*membrane*_ are indeed more than 10% lower at 4 K than at 300 K. Therefore, we conclude that the reduction in thermal conductivity in NWs at 4 K is partially caused by the changes in the phonon dispersion. Furthermore, we observe that the width dependence of the thermal conductivity is steeper at 4 K. Two mechanisms can explain this. First, the presence of internal scattering at 300 K, whose impact increases with width, flattens the width dependence of thermal conductivity at room temperature. Conversely, the absence of such internal scattering at 4 K partially explains the observed trend. Second, as the width dependence of thermal conductance calculated from the phonon dispersion is larger than the change in cross section, it additionally contributes to the slope increase at 4 K. Given our experimental conditions, it is also interesting to compare the thermal conductance of our NWs that display ballistic transport to the quantum of thermal conductance 
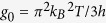
. For NWs with a length of 0.5 μm (2 μm), the thermal conductance is approximately 100 *g*_0_ (40 *g*_0_), showing that a large number of modes are populated, agreeing with what is expected in NWs of such dimensions^46^.

## Conclusion

We have investigated different heat transport regimes in silicon NWs at different temperatures. The measured values of thermal conductivity are in good agreement with the experimental and theoretical results from the literature in the 10–300 K range. At room temperature, the thermal conductivity is independent of the NW length, representing the purely diffusive heat transport regime. However, at 4 K, we observed a transition from the diffusive to ballistic regime in short NWs. This semi-ballistic regime stems from the absence of internal scattering processes and partially specular surface scattering. Next, we compared the phonon dispersion in a simple membrane and NWs of different widths and demonstrated that the thermal conductance is decreased in NWs as the width is reduced. This has been confirmed experimentally at 4 K, where the modified NW phonon dispersion was used, showing that it is possible to observe an impeded heat flux due to a modification of the phonon dispersion at cryogenic temperatures. The semi-ballistic phonon transport presented in this work, as well as the experimental observation of the phonon dispersion impact on thermal conductivity, opens new possibilities toward heat conduction engineering in periodic nanostructures fabricated by the top-down approach. This is because it is possible to design them with a broad range of wave properties, including bandgaps.

## Additional Information

**How to cite this article**: Maire, J. *et al*. Ballistic thermal transport in silicon nanowires. *Sci. Rep.* **7**, 41794; doi: 10.1038/srep41794 (2017).

**Publisher's note:** Springer Nature remains neutral with regard to jurisdictional claims in published maps and institutional affiliations.
